# Guilt emotion and decision-making under uncertainty

**DOI:** 10.3389/fpsyg.2025.1518752

**Published:** 2025-03-28

**Authors:** Amelia Gangemi, Chiara Rizzotto, Febronia Riggio, Margherita Dahò, Francesco Mancini

**Affiliations:** ^1^Department of Cognitive Sciences (COSPECS), University of Messina, Messina, Italy; ^2^Department of Psychology, Educational Science and Human Movement (SPPEFF), University of Palermo, Palermo, Italy; ^3^Associazione di Psicologia Cognitiva, Viale Castro Pretorio, Rome, Italy

**Keywords:** moral emotions, guilt, anger, decision under uncertainty, framing effect

## Abstract

This paper examines the impact of moral emotions, such as guilt, on decisions under risk. In two experiments, we demonstrated that guilt emotion influences preferences for risky and riskless choices, depending on the subject’s moral goal, i.e., reparation or expiation, whereas anger consistently elicits a preference for taking risks. Unlike other moral emotions (e.g., anger), guilt is thus not characterized by a fixed preference for either risky or riskless choices. Preferences vary as a function of the option that may satisfy the moral goal, instead of by a form of bias that the different emotions play toward decisions under risk. Finally, in both experiments, responses appear to be based on the framing of the decision problem according to the induced emotional state (guilt or anger), rather than on the descriptions of the outcomes as given in the options (gain-loss framing effect).

## 1 Introduction

Decision-making under risk is mainly a cognitive field of research. Since the end of the last century, decision researchers have begun incorporating emotions into their studies (see Smith and Ellsworth, 1985; Brickhouse and Smith, 2015). Once they recognized the strong influence of emotions on decision-making, they turned their attention to the role of emotions in decision-making under uncertainty. Although the specific role of moral emotions (e.g., regret and guilt) in shaping decisions across various domains, such as economics (see Gurevich et al., 2012) has been extensively studied (e.g., Weiner, 1985; Smith and Ellsworth, 1985), this field remains far from complete. With this paper, we aim to contribute to this research area by examining the influence of a specific moral emotion—guilt—on decision-making under risk.

### 1.1 Valence-based approach vs. appraisal-tendency approach to emotions and decisions

Initially, research on how emotions affect decision-making processes followed a valence-based approach, contrasting the effects of positive versus negative emotional states on judgment and choice (e.g., Johnson and Tversky, 1983; Schwarz and Clore, 1983; Damacio, 1994). However, these studies did not specifically investigate whether and how different emotions of the same valence (e.g., fear and anger) differentially affect decision making (cf. Lerner and Keltner, 2000; Ellemers et al., 2019). Only later have studies systematically examined the influences of specific emotions on judgment and choice (e.g., Bodenhausen et al., 1994; Keltner et al., 1993). Indeed, according to the appraisal tendency approach (see Lerner and Keltner, 2000; Lerner et al., 2015), several authors have argued that each emotion carries with it a tendency to perceive new events and objects in a way that is consistent with the original cognitive appraisal dimensions of the emotion.

In line with this approach, specific predictions have been made about how and when different emotions influence choice. For example, Lerner and Keltner (2000, 2001) examined the influence of two emotions of equal valence but different nature (anger and fear) on risk perception. The authors showed that fear and anger have opposite effects on risk assessment. Fearful people made pessimistic judgments about future events and were risk-averse, whereas angry people made optimistic judgments and were risk-seeking.

This appraisal hypothesis appears to be supported by recent studies in the field of affective neuroscience (e.g., Ma et al., 2017). A number of lesional, clinical and neuroimaging studies confirm the existence of distinct, though often overlapping, neural circuits for emotions such as happiness, anger, sadness and fear. The differences in the networks involved are not only between emotions of different valence (e.g., joy and sadness), but also between emotions of the same negative valence (e.g., anger, fear, or sadness). This type of evidence further justifies the hypothesis that emotions of the same (negative) valence can trigger different cognitive dispositions, with some strategy specific activation.

### 1.2 Moral emotions

Despite the growing interest in the influence of specific emotions on judgment and choice, little attention has been paid to the influence of moral emotions (e.g., shame, regret, guilt, and anger) on the same cognitive processes (for notable exceptions, see Mellers et al., 1999), even though these emotions are involved in numerous judgments and decisions in everyday life and influence choices. In recent decades, a growing body of research has finally brought to light the importance of these emotions in the decision-making process. A diverse mix of emotions, including anger, guilt, shame, contempt, empathy, gratitude, and disgust, have been suggested as critical factors in this process (Fitouchi et al., 2022). Since the publication of “The Moral Emotions” chapter by Haidt (2003), these emotions have been defined as those “*that are linked to the interests or benefits of others welfare either of society as a whole or at least of persons other than the judge or agent*.” Accordingly, they provide an emotional moral signal that indicates a change in moral acceptability, associating aversive emotions with sin, transgression and wrongdoing, and positive emotions with moral behavior. Individuals can even anticipate the emotional outcomes of their actions, which can strongly influence their moral behavior (Tangney et al., 2007). Moral emotions thus place a person in a motivational state in which there is an increased tendency to engage in certain goal-related actions (i.e., prosocial action tendencies), such as expiation and consolation, in response to eliciting events (i.e., triggers) (cf. Haidt, 2003; Lerner et al., 2015). Thus, one of the main functions of moral emotions is to regulate social behavior, often in terms of the long-term interests of a social group or of the individual to be socially accepted, rather than the short-term interests of the individual. Anger, for example, is generally said to be a response to goal blockage and frustration caused by unjustified insults and reactions (Berkowitz and Harmon-Jones, 2004; Berkowitz, 2012). It generally motivates one to right wrongs by attacking, humiliating, or getting back at the person who is perceived to have acted unfairly or immorally. The readiness to attack or fight manifests itself not only experientially but also physiologically. For example, anger is associated with neural activation features of approach motivation and sometimes with changes in peripheral physiology that may prepare one to fight, such as increased blood flow to the hands (Harmon-Jones and Sigelman, 2001; Berkowitz, 2012). In contrast, guilt is thought to be triggered by the violation of moral rules and imperatives (cf. Freud, 1930; Lazarus, 1991), especially when these violations cause harm or suffering to others (Horne and Powell, 2016). However, guilt is most strongly triggered when one’s harmful action also threatens one’s communion or kinship with the victim. In line with this view, guilt motivates the offender to help the victim or to make amends for the transgression (Jonas et al., 2022), even if this is against the offender’s utility. It also triggers the goal of making amends, preventing further guilt, and expiation to restore or improve relationships (Mancini and Gangemi, 2021; Gangemi et al., 2021).

At the same time, however, if it exceeds certain levels, becoming overwhelming, guilt triggers self-defensive responses that hinder reparative behaviors (for example, make amends for the transgression), such as the desperate search for justifications (Giner-Sorolla, 2013). In summary, guilt can be viewed as a “self-condemning” moral emotion that informs self-views and guides reparative behaviors (Ellemers et al., 2019). Therefore, according to this framework, guilt would influence decision making under uncertainty to increase the likelihood of achieving these goals, to repair or expiate. It is precisely this guilt emotion (and not the pathological or disruptive ones) that we will focus on this paper.

### 1.3 What factors influence decisions under uncertainty?

Traditionally, risk aversion and risk seeking have been explained by invoking the framing effect (Kahneman and Tversky, 1979; Kahneman and Tversky, 1981, 1984; for recent review: Fisher and Mandel, 2021). In their studies on decisions under risk, Kahneman and Tversky examined the effects that alternative descriptions or “frames” can have on decision-making, particularly how framing influences preferences and choices. Consider Kahneman and Tversky’s (1981) widely used “Asian disease problem” (ADP). In a within-subject design, participants are asked to imagine that the United States is preparing for the outbreak of an unusual Asian disease that is expected to kill 600 people. Two alternative programs are proposed to combat the disease (A and B). Under the gain frame, participants read that the exact scientific estimates of the consequences are as follows: “If Program A is adopted, 200 people will be saved. If Program B is adopted, there is a 1/3 probability that 600 people will be saved and a 2/3 probability that no one will be saved.” Under the loss frame, participants learn that “if Program C is adopted, 400 people will die. If Program D is adopted, there is a 1/3 probability that nobody will die and a 2/3 probability that 600 people will die.” Although a preference for the certain “risk-averse” option (Program A under the gain frame) should lead a subject to prefer the equivalent option under the loss frame (Program C), the typical choice for people is to select A under gain frames and D under loss frames. Across investigations, an average of 70–80% of respondents become risk-seeking (i.e., choose the gamble) when the choices are framed as losses, and become risk averse (i.e., choose the certain outcome) when identical choices are framed as gains. In summary, there is a certainty effect, whereby certain gains are sought, and certain losses are avoided. According to this theory, framing effects can be exceptionally large, reliable (Gosling et al., 2020; Fisher and Mandel, 2021), and generalizable across individuals, overwhelming any individual differences in risk attitudes. However, some evidence suggests otherwise. Indeed, small and seemingly unimportant changes in the wording of options in the ADP have eliminated or even reversed the framing effect. For example, options can be framed in a way that conveys mixed outcome valences, such as stating that “200 people will be saved and 400 people will die” (Kühberger and Tanner, 2010; Mandel, 2001). More recently, Tombu and Mandel (2015) introduced the “Explicated Valence Account” (EVA), according to which framing effects may also depend on the overall positive or negative valence of the description of each option. For example, in the ADP perspective, “200 lives will be saved” has positive explicated valence because saving lives, which is explicitly mentioned, is a positive outcome. However, “200 people will not die” also has positive explicated valence even though the term die (a negative descriptor) is used to describe the option. In two experiments, the authors pitted EVA against prospect theory by creating situations in which frame and explicated valence make opposite predictions. They found that preference reversals were influenced by changes in explicated valence, and not by contradictory risk attitudes across gain and loss domains. Indeed, in their experiments, perceived risk attitudes were stable across frames. Another study that contradicts the gain-loss framing effect is that of Lerner and Keltner (2001). They showed that individual differences in emotions influence outcomes and that these influences hold across gain-loss framing conditions. For example, the sense of safety and control associated with anger leads angry individuals to make risky choices across frames, whereas the sense of uncertainty and lack of control associated with fear leads fearful individuals to make risk-averse choices across frames.

Consistent with these findings are data from a recent systematic review and meta-analysis (Bartholomeyczik et al., 2022) of 28 experiments. They support the hypothesis that the gain-loss /valence framing effect does not influence decision-making under risk and uncertainty. In line with this review, a recent meta-analysis on if and how *moral* individuals’ choices differences are affected by in framing presentations (e.g., gain vs. loss). McDonald and colleagues (McDonald et al., 2021) found that the role of gain-loss/valence framing presentation even disappears when moral demands are present. In addition, Kartin and Murniati (2018) show that responsibility for risk decisions influences decision preferences; gain-loss/valence framing has no effect on individuals who were burdened with responsibility within a group.

### 1.4 Study objectives

In line with the framework presented so far, we examined whether and how guilt emotion influences decisions under risk. Due to its behavioral specificity and self-condemning tendencies (e.g., Haidt, 2003; Mancini et al., 2008; Giner-Sorolla, 2013; Ellemers et al., 2019), we hypothesized that the influence of guilt on individuals’ decisions would lead them to make the choice (risky or riskless) that allows them to pursue the moral goal of repairing the harm caused to the victim (see Mancini and Gangemi, 2021), or atoning for the offense. According to the Dobby effect (Nelissen and Zeelenberg, 2009), guilty people punish themselves if they have no opportunity for compensating the victim of their transgression. In other words, individuals’ risky or riskless choices would vary as a function of the goals activated by guilt, i.e., repair and expiation. To test this hypothesis, two different experiments were conducted. In the first, the question options were formulated in a specific way: the only option that allowed our guilty subject to pursue the goals activated by guilt was the riskless one. Thus, our prediction was that our guilty subjects would prefer the riskless choice. Second, to ensure that the results of Experiment 1 were not due to general risk aversion but to a specific moral goal, we varied the response alternatives to obtain an option that was both risky and carried the moral goals of restoring justice for the guilty subjects. We expected that participants who felt guilty would continue to prefer the choice (risky or riskless) that allowed them to correct or expiate the wrong.

## 2 Methods

### 2.1 Experiment 1

With this study, we wanted to show that the moral goal of atonement or rectification due to guilt would lead subjects to make the choice (risky or riskless) that would allow them to pursue these goals. In the present study, the option that allowed guilty subjects to expiate, prevent further guilt, or rectify was the riskless one. Since we were looking at choices with a monetary penalty, the riskless choice was indeed the one that allowed payment to be made with certainty. In contrast, the risky choice did not allow any payment to be made. To ensure that any effects were indeed due to guilt and not to negative emotions in general, participants were assigned to one of two emotion induction conditions: guilt and anger. The choice of anger as a negative moral emotion to contrast with guilt was based on the fact that people can feel anger when they see themselves as victims of an offense. Participants wrote about a guilt-related or an anger-related life event to evoke the emotion. The emotional states were therefore neither induced by nor related to the task used later in the study. We predicted that the preferences of participants assigned to the guilt-induction condition would be risk-averse, regardless of whether the questions were framed as gains or losses (formulation effect). In contrast, according to the literature (see Lerner and Keltner, 2000, 2001), the preferences of participants assigned to the anger-induction condition would be risk-seeking, regardless of whether the questions were framed as gains or losses. In this study, we considered choices with a pecuniary penalty imposed on the individual. We expected that individuals faced with risky decisions would take into account not only the expectations of monetary outcomes but also the moral implications of these outcomes.

#### 2.1.1 Participants and design

One hundred and seventy-six undergraduate students (96 female, 80 male) from the University of Messina completed the study for course credit. Their mean age was 22 years, with a range of 19–42 years. The participants were randomly divided into four independent groups, which were well-balanced in terms of age and gender. Each group received one of four versions of the decision problem (see Table 1). A 2 (emotional state induction: guilt or anger) × 2 (framing: gain or loss) between-subjects design was implemented to test the hypotheses.

**Table 1:** Percentages (and frequencies) of responses across the four conditions of Experiment 1 (*N* = 176).

Emotion						
	Anger			Guilt		
Question option	*n*	Risk-seeking	Risk-aversion	*n*	Risk-seeking	Risk-aversion
Gain	40	60 (24)	40 (16)	48	25 (12)	75 (36)
Loss	40	80 (32)	20 (8)	48	19 (9)	81 (39)

Participants signed informed consent before participating in the experiment.

#### 2.1.2 Materials and procedure

Following the procedure used by Schwarz and Clore (1983), emotional state was manipulated by asking participants to describe either a guilt-related or an anger-related personal life event. Participants were asked to describe a guilty (or angry) event in their recent life as vividly as possible, including details of what they were feeling and thinking. They were told that they would have 15 min to remember and write the stories. After writing the event, part 1 of a manipulation check questionnaire was administered to check the effectiveness of the induction. Participants were asked how guilty/angry they felt after describing the event. Individuals rated their feelings of guilt and anger on a scale from 0 to 100, with anchors at 0 (not at all guilty/angry) and 100 (totally guilty/angry).

##### 2.1.2.1 The decision problem

The participants were then given the decision problem, in which they were not told whether they were the offenders or the victims with regard to the fine. The decision problem was as follows (translated from Italian): *When you come back home, you find a €1,200 speeding fine.* The question format with gain framing was:

- if you pay the fine immediately, you will save €400.
- if you appeal the fine, there is a one-third probability that you will save €1,200 and a two-thirds probability that you will save nothing.

In the loss framing format, these sentences are:

- if you pay directly, you will pay €800.
- if you appeal the verdict, there is a one-third probability that you will pay nothing and a two-thirds probability that you will pay €1,200.

In all conditions, participants were told to read the question options (regarding the decision problem) and to indicate which alternative they chose (a or b), taking as much time as they needed. The expectations of the monetary outcomes in the decision problems were indistinguishable. The order of the two different options was randomized.

##### 2.1.2.2 Manipulation check questionnaire

After completing the decision task, all participants were asked to complete three-item Manipulation Check Questionnaire on guilt about the penalty in the task (How guilt did you feel after reading the problem? How guilt did you feel about the penalty?) and the fairness of the penalty (How far is the penalty?). Individuals rated their feelings of guilt (two items) and the fairness of the penalty (one item) by marking visual analog scales (VAS) as follows: ratings of guilt were made on a range of 0 to 100, with anchors at 0 (not at all guilty) and 100 (totally guilty); ratings of the fairness of the penalty were made on a range of 0 to 100, with anchors at 0 (not at all fair) and 100 (totally fair). If the manipulation was effective “guilty” participants would report feeling more guilt during the task and from the fine, and would rate it as fairer than the “angry” group.

#### 2.1.3 Results

##### 2.1.3.1 Manipulation check questionnaire

We analyzed the data for guilt felt after the emotional state induction (Manipulation Check Questionnaire, Part 1) using univariate ANOVA. Results revealed that the induction was effective. After writing about the past life event involving guilt, individuals perceived more guilt (*M* = 61.13, *SD* = 17.56) than individuals in the angry condition (*M* = 38.37, *SD* = 20.21) (*F* (1, 169) = 62.05, *p* < 0.001). By contrast, after writing about the past life event involving anger, individuals perceived more anger (*M* = 62.14, *SD* = 18.2) than individuals in the guilty condition (*M* = 37.97, *SD* = 19.08) (*F* (1,169) = 69.47, *p* < 0.001). For manipulation check variables completed by participants immediately after the decisional task Manipulation Check Questionnaire, Part 2, the manipulation of emotional states again was effective (*F* (1, 169) = 49.87, *p* < 0.001). Guilty participants perceived more guilt arising from the fine (*M* = 62.75, *SD* = 25.66) than angry participants (*M* = 38.8, *SD* = 17.16). Moreover, guilty participants evaluated the penalty as fairer (*M* = 63, *SD* = 17.28) than angry participants (*M* = 37.56, *SD* = 19.93) (*F* (1, 169) = 77.79, *p* < 0.001).

##### 2.1.3.2 The decision problem

We examined the effect of our independent variables (“emotional state induction” versus “question option format”) on participants’ choices, testing a logistic regression model of participants’ choices.

The predictors included “emotion-induction condition,” “question option format,” and their interaction. The model was statistically significant, χ^2^ (3, *N* = 176) = 47.08; *p* < 0.001. As expected, Wald’s statistics revealed that only the “emotion-induction condition” predictor was significant (*p* < 0.001). Participants’ risk-aversion responses occurred much more often when individuals felt guilty (78%) (cf. Table 1). By contrast, risk-seeking responses occurred much more often in the anger-induction condition (70%). The other two predictors (“question option format” and “emotion- induction condition” and “question option format” interaction) were not significant.

#### 2.1.4 Discussion

The results of Experiment 1 support our predictions. The emotion induction condition appears to be a determinant of individuals’ preferences (risk-seeking or risk-averse), regardless of the format of the question options (the gain-loss framing effect invoked by Kahneman and Tversky, 1981). Our participants’ responses appear to be based on the framing of the decision problem according to the induced emotional state (guilt or anger), rather than on the descriptions of the outcomes as given in the options. Our participants preferred the risky or riskless choice over the gain-loss formulation effect invoked by Tversky and Kahneman when trying to restore justice, either because they felt themselves to be the offenders (guilt induced condition) or the victims of an offense (anger induced condition). Our explanation for why “guilty” participants tend to choose the riskless option is that feelings of guilt lead them to believe they deserve the fine. This belief leads them to think that chance could favor them, prompting them to choose the riskless option to avoid the possibility of a unexpectedly favorable outcome, such as saving money. By contrast, “angry” subjects may feel that the fine is unjustified and that they do not deserve it. This could lead them to believe that chance might favor them, increasing their expectation of a favorable outcome, such as saving money. This belief motivates them to prefer the risky option. It is worth noting that in the present study, participants’ moral emotions were directly and intentionally manipulated, and thus the emotional states were unrelated to the event being evaluated.

### 2.2 Experiment 2

Although the results of the previous experiment support the prediction that participants’ preferences vary as a function of the induced moral emotion (guilt vs. anger), an important issue may remain unresolved in this experiment. There is no guarantee that the induction of guilt resulted in riskless preference due to the specific moral goal of atonement or restitution, as opposed to a general risk aversion. In the earlier experiment, the question options were formulated in a specific way: the only option that allowed our guilty subject to repair the offense was the riskless one. We did not vary the answer alternatives in order to obtain an option that was both risky and had moral weight for the guilty subjects. On this basis, there could be alternative explanations for our results that do not take into account the moral value of the choice. Instead, in our experiment, a general risk aversion effect, which is also present for other emotions such as fear (see Lerner and Keltner, 2000, 2001), may have influenced the decisions of participants in the guilt-induction group. Experiment 2 was therefore conducted to clarify whether the risk-averse choices of guilty participants, observed in Experiment 1, were solely due to a general risk aversion associated with the emotion of guilt, or whether the non-risky choice was instead driven by a moral goal—namely, to correct or atone for the wrong and avoiding further guilt. The latter could arise from selecting the risky option that provided the possibility of paying nothing. Experiment 2 was to some extent a replication of Experiment 1, but the experimental task was partly modified in order to show that our results were due to a specific moral goal effect of guilt, and thus to remove the confusion between the moral value of the choice and the general risk aversion effect.

To this aim we used 8 different problems. The problems were divided into the “role of the decision maker” (offender/victim), the “role of the person paying for the decision” (offender/victim) and the question-option format” (gain/loss). In this way, unlike the earlier experiment, in the problems, we used also options that were both risky and had the moral goal of restoring justice for the guilt-induction group. In particular, by varying the role paying for the choice (offender/victim), both risky and riskless options could allow for restitution or expiation for participants in the guilt-induction group. In fact, if the role paying for the decision was the “victim,” then the risky choice was the one that would allow participants in the guilt induction condition to restore justice: it gave the victim a chance to obtain justice, i.e., not to pay at all. If the role paying for the decision was “guilty,” then the riskless choice was the one that would allow participants in the guilt-induction condition to re-establish justice: it made the guilty pay with certainty. The riskless choice would instead allow him/her to pay nothing.

Thus, in line with the results of a preliminary study (Mancini and Gangemi, 2006), we expected that participants who felt guilty would prefer the choice (in the current experiment, risky or riskless) that allowed them to correct or atone for the wrong, as a function of the role paying for the decision (offender or victim) and independent of both the role making the choice (offender or victim) and the option format of the question (gain or loss). In line with the literature (e.g., Lerner and Keltner, 2001), we predict that angry participants will prefer the risky choice regardless of who makes or pays for their choice and regardless of the option format of the question, and that control participants will follow the gain-loss formulation effect regardless of who makes or pays for their choice. To test these hypotheses, we examined three groups of volunteers assigned to three different emotion induction conditions (guilt vs. anger vs. neutral) instead of the two groups of Experiment 1 (guilt vs. anger). In addition, to assess the effectiveness of the emotion induction manipulation, we included a measure of state guilt, as assessed by the Guilt Inventory (Jones and Kugler, 1993; Jones et al., 2000), and state anger, as assessed by the State–Trait Anger Expression Inventory (Spielberger, 2010). Finally, we included a measure of negative affect (Positive and Negative Affect Scale, PANAS; cf. Watson et al., 1988) to ensure that (a) the manipulation of affect in the two negative affect induction groups (guilt vs. anger) would result in negative affect, and (b) any differences in decisions made between these two groups would be due to a specific emotional effect. Following Lerner and Keltner (2001), for each problem and each set of alternatives, participants indicated the extent to which they would prefer one option over the other, if at all.

#### 2.2.1 Participants and design

Three hundred and thirty-four undergraduate students (189 female, 145 male) from the University of Messina participated for course credit. Their mean age was 28.8 years, ranging from 18 to 49 years. The design was 3 × 2 × 2 × 2 independent groups with the factors: “emotional state induction” (guilt, anger or neutral), “role making the choice” (perpetrator or victim), “role paying for the choice” (perpetrator or victim) and “question option format” (gain or loss).

Participants signed informed consent before participating in the experiment.

#### 2.2.2 Materials and procedure

Participants were tested in six independent groups (consisting of approximately 55 individuals), each of which received one of the 8 versions of the decision problem (see Table 2). At the beginning of the session they were given the State Guilt Inventory, the State–Trait Anger Expression Inventory (Spielberger, 2010) and the PANAS scales (see below), a booklet with written instructions and a decision problem (see below).

**Table 2:** Mean affect ratings, standard deviations and F-ratio by the three affect induction groups (guilt, anger, neutral), before and after the affect induction.

	Guilt ratings		F ratio, df, *p*-value	Anger ratings		F ratio, df, *p*-value	Negative affect ratings		F ratio, df, *p*-value
	Pre-affect induction	Post-affect induction		Pre-affect induction	Post-affect induction		Pre-affect induction	Post-affect induction	
Guilt-group (*n* = 117)	25.7 (5.7)	30.4 (7.4)	*F* (2,319) = 38.4 *p-*value = 0.001**	13.5 (5.3)	13.62 (5.1)	*F* (2,318) = 7.37 *p*-value = 0.001**	17.4 (6.4)	24 (7)	*F* (2,331) = 46.1 *p*-value = 0.001**
Anger-group (*n* = 107)	26.1 (7.3)	25.2 (7)		13.7 (6.1)	16 (8.1)		15.6 (5.2)	24.5 (9.8)	
Neutral-group (*n* = 110)	25.6 (4.5)	22.6 (5.6)		13.1 (5.8)	13.12 (5.2)		17.1 (5.8)	16.9 (6.3)	

##### 2.2.2.1 Baseline emotion

We assessed baseline differences in guilt, anger, and negative affect by asking participants to complete three different questionnaires at the beginning of the experiment. Specifically, to assess current levels of guilt, we used the 10-item State Guilt Inventory (Jones and Kugler, 1993; Jones et al., 2000). Responses were given on a 5-point scale, with a low score indicating strong disagreement and a high score indicating strong agreement. The subscale was averaged to produce a reliable scale (α = 0.83). The total score (range 10–50) was calculated by summing the scores of the 10 items. Items were coded so that higher scores reflected greater state guilt.

The State–Trait Anger Expression Inventory, STAXI (Spielberger, 2010) was used to assess current levels of anger. Responses were given on a 4-point scale (from “almost never” to “almost always”). The items were chosen to characterize the current experience of angry feelings. The interna consistency (alpha coefficients) of the scales is quite high 0.93. The total score (range 10–40) was calculated by adding the scores of the 10 items. The items were coded so that higher scores reflected greater state anger.

Finally, we assessed baseline negative affect using the Positive and Negative Affect Scale (PANAS) (Watson et al., 1988), which consists of 20 emotion terms on which participants indicate their current feelings (1 = “very little or not at all”, 5 = “extremely”). These 20 items are grouped into two subsets, one measuring positive affect and one measuring negative affect, and both subsets were averaged to form reliable scales (α = 0.73 and 0.88, respectively). We combined all 10 negative items from the PANAS into a negative affect factor (eigenvalue = 5.78, 48% of variance explained). Using principal component analysis, we also combined two anxiety-related items from the PANAS (“jittery” and “nervous”) into an “anxiety factor” (eigenvalue = 2.15, 72% of variance explained).

##### 2.2.2.2 Emotion induction

The materials and procedure were the same as in Experiment 1. The only exception was the control group, in which emotional state was manipulated by having participants describe a neutral personal life event. At the end of the affect induction period, participants were again asked to complete the State Guilt Inventory (to quantify guilt induction through the State Guilt Inventory total score), the State Trait Anger Expression Inventory (to quantify anger induction through the State Trait Anger Expression Inventory total score), and the PANAS scales (to quantify negative emotional impact through the Negative Emotion Factor score).

##### 2.2.2.3 The decision problem

All participants then read one of the 8 problems with a story describing situations in which a builder and an architect were fined for building a house. The problems were divided into the “role of the decision-maker” (offender/victim), the “role of the person paying for the decision” (offender/victim) and the “question-option format” (gain/loss), as follows (translated from Italian):

The problem where the “offender” is the character who makes the choice and the “victim” pays for that choice:

*Imagine that you are the owner of a construction company and that a random check by the police reveals a violation of safety regulations on a site where you are working. You are to blame because you did not take the necessary precautions. The architect, in his capacity as site manager, had warned you of the safety measures to be taken, but you negligently failed to take them into account. According to the law, the responsibility lies with the architect, so he is suspended from the association for 3 months. For the same reason, the architect is liable for a fine of €30,000. For legal reasons, only you can decide to appeal.*

*Your lawyer will inform you of this:*

- *If you do not appeal, the architect will pay €20,000.*
- *If you do appeal, the architect has a 2/3 chance of paying the full €30,000 and a 1/3 chance of paying nothing.*

The problem where the “offender” makes the choice and pays for it:

*Imagine that you are the owner of a construction company and that a random police check on one of your sites reveals a breach of safety regulations. You are to blame because you did not take the necessary precautions. The architect, in his capacity as site manager, had warned you of the safety measures to be taken, but you negligently failed to take them into account. According to the law, the responsibility lies with the architect, so he will be suspended from the association for 3 months. However, you have to pay a fine of €30,000. You decide to appeal.*

*Your lawyer informs you that.*

- *If you do not appeal, you will have to pay €20,000.*
- *If you appeal, there is a 2/3 chance that you will have to pay €30,000 and a 1/3 chance that you will have to pay nothing.*

In all problems, for each set of alternatives, participants indicated to what extent, if at all, they would prefer one option over the (see Lerner and Keltner, 2001). The response options ranged from 1 (“very much prefer option A”) to 7 (“very much prefer option B”). The expected monetary outcomes in the decision problems were indistinguishable. The order of the two different options was randomized.

#### 2.2.3 Results

##### 2.2.3.1 Manipulation check: measures of mood induction

Table 2 shows the mean emotion ratings on scales of guilt, anger, and negative affect for participants in all three emotion-induction conditions both before and after the emotion-induction procedure. Each measure was subjected to a 2 × 3 ANOVA comparing “time” (before vs. after) as a within-group factor and “emotion-induction condition” (guilt, anger, or neutral) as a between-group factor.

For the state “guilt,” a significant “time” × “emotion-induction condition” interaction was found, *F* (2, 319) = 38.4, *p* < 0.001. The nature of the interaction was analyzed by studying which groups displayed a significant pre-to-post increase in state guilt. The increase in the guilt-induction group was significant (*t* (117) = 8.9, *p* < 0.001), but no significant effect was found in the anger-induction group (*t* (105) = 0.96, *ns*). In the neutral group, a pre-to-post decrease in state guilt was found (*t* (100) = 7.6, *p* < 0.001). Thus, the manipulation was successful overall in inducing the relevant emotion. For the anger measure, there was a significant “time” × “emotion-induction condition” interaction, *F* (2, 318) = 7.37, *p* < 0.001. The nature of the interaction was analyzed by studying what groups displayed a significant pre-to-post increase in anger. A significant pre-to-post increase in anger was found in the anger-induction group (*t* (99) = 3, *p* < 0.05). No significant effects were found both in the guilt-induction group (*t* (117) = 0.4, *ns*) and the neutral group (*t* (105) = 0.1, *ns*).

Finally, in the case of the negative emotion measure, there was a significant “time” × “emotion-induction condition” interaction was found, *F* (2, 331) = 46.1, *p* < 0.001. Both the anger-induction group (*t* (106) = 11.35, *p* < 0.001) and the guilt-induction group (*t* (116) = 10.19, *p* < 0.001) displayed a significant pre-to-post increase in negative emotion. No significant effect was found in the neutral group (*t* (109) = 0.23, *ns*).

Therefore, it seems that the experimental affect manipulation was successful. The guilt induction led to an increase in state guilt, whereas the other manipulations did not. Similarly, the anger induction led to increases in anger, whereas no increases in anger were observed in the other conditions. Finally, negative emotions increased in both the guilt and anger induction groups, but not in the control group.

##### 2.2.3.2 The decision problem

Table 3 shows the mean ratings of participants’ preferences across all experimental conditions. Each measure was subjected to a 3 × 2 × 2 × 2 ANOVA with “emotion-induction condition” (guilt, anger, or neutral), “role choosing” (offender/victim), “role paying for the choice” (offender/victim), and “question option format” (gain/loss) as between-group factors. As predicted, significant “emotion induction” × “the role paying for the choice” interaction effects were found (*F* (2, 310) = 10.3, *p* < 0.001). Individuals in the guilt-induction group favored the riskless choice when the offender was paying for it, reporting lower ratings (*M* = 2.6, SD = 1.9) than participants in the anger- (*M* = 4, SD = 2.2, *t* (112) = 5.35, *p* < 0.001) and neutral- (*M* = 3.8, SD = 2, *t* (108) = 4.9, *p* < 0.001) induction groups. The experimental manipulations had no effect on participants’ preferences when the victim paid for the choice. Indeed, none of the between-group comparisons in this condition reached significance (*t =* 1.4*, ns*), indicating that all groups, including the guilt-induction group, tended to prefer the risky choice. Significant “emotion induction” × “option interaction” effects were also found (*F* (2, 310) = 4.08, *p* < 0.05). According to the literature, participants in the neutral-induction condition tend to favor the risky choice if the option was formulated as a loss reporting higher ratings (*M* = 4.12, SD = 1.9) than when the option was formulated as a gain (*M* = 3.48, SD = 1.8, *t* (104) = 1.8, *p* < 0.05). In the other two emotion-induction conditions, the formulation effect by Kahneman and Tversky (1981) was not present (*t*s < 1.3). The other interactions were not significant.

**Table 3:** Mean ratings of risky choices across all conditions of Experiment 2 (*N* = 334).

S/he who					Pays	
			Guilty		Victim	
Emotions	S/he who chooses	Options	*n*	*M* (*SD*)	*n*	*M* (*SD*)
Guilt	Offender	Gain	18	2.6 (1.9)	14	4.5 (2.1)
		Loss	10	1.3 (0.5)	13	4.2 (1.9)
	Victim	Gain	9	2.6 (2.3)	11	4.8 (2.5)
		Loss	15	1.5 (1.1)	20	4.5 (1.9)
Anger	offender	Gain	14	3.6 (2.5)	19	4 (2.2)
		Loss	18	3.6 (2.1)	8	4.4 (2.7)
	Victim	Gain	12	4.5 (1.9)	15	5.2 (2.2)
		Loss	13	4 (2.1)	10	4.3 (3.1)
Neutral	Offender	Gain	14	3.4 (1.8)	11	3.8 (2)
		Loss	20	4.1 (2.1)	8	4.9 (1.7)
	Victim	Gain	18	3.7 (2.2)	17	3.4 (1.6)
		Loss	11	4 (2.2)	16	3.8 (1.4)

#### 2.2.4 Discussion

The results of Experiment 2 support our predictions. The “emotion induction condition” and the “role paying for the choice” factors appear to be determinants of individuals’ preferences (risk-seeking or risk-averse), regardless of the loss-gain format of the question options (the framing effect invoked by Kahneman and Tversky, 1981) and whether the decision was made for oneself or for another person (i.e., whether the decision-maker was personally involved or not). Participants who felt guilty continued to prefer the choice (risky or riskless) that allowed them to correct or expiate the wrong. Their preference for the risky or riskless choice depended on who paid for the choice: if the “victim” paid, then the risky choice; if the “offender” paid, then the riskless choice, regardless of who made the choice.

Our explanation is that by varying the role responsible for the payment (offender/victim), both the risky and riskless options could serve as means of restitution or expiation for participants in the guilt induction group. Specifically, if the role responsible for the payment was the “victim,” then the risky choice allowed participants in the guilt-induction condition to restore justice, as it provided the victim with a chance to avoid payment altogether. Conversely, if the role responsible for the payment was the “guilty” party, then the riskless choice enabled participants in the guilt-induction condition to re-establish justice, ensuring that the guilty party paid with certainty. In contrast, the risky choice could result in the guilty party paying nothing at all. Furthermore, in line with the literature, angry participants tended to favor the risky choice, regardless who chose or paid for it. “Angry” subjects might feel that the fine is unjustified in any case and that those who have to pay it (offender or victim) do not deserve it. This perception could lead them to believe that the risky choice is the only one that could restore justice as it provided the victim with a chance to avoid payment altogether. As predicted, Kahneman and Tversky’s (1981) loss-gain formulation effect was only present in our control condition.

In the present study, participants’ moral emotions were directly manipulated, and thus emotional states were independent of the event being evaluated, the task.

## 3 General discussion

Our hypotheses were supported by the results of both experiments. Guilt seems to be an important determinant of individual preferences (risk-seeking or risk-averse). This moral emotion seems to guide (risky or riskless) decisions in the attempt to pursue the moral goal of restoring justice by repairing the harm caused to the victim or atoning for the offense (e.g., Ellemers et al., 2019; Giner-Sorolla, 2013). In Experiment 1, our guilty subjects preferred the riskless choice, the only option that allowed them to make amends for the offense. In Experiment 2, to show that the results of the earlier experiment were not due to a general risk aversion effect, but to a specific moral goal effect, we varied the role paying for the decision. Participants who felt guilty still preferred the choice (risky or riskless) that allowed them to restore justice. If the victim paid for the choice, then we observed a risky choice (i.e., the one that might give the victim a chance to get justice, not paying at all). If the offender paid, then there was a riskless choice (i.e., the one that made the offender pay with certainty). Finally, we showed that in both experiments, participants’ responses appear to be based on the framing of the decision problem according to the guilt emotional state, rather than on the descriptions of the outcomes as given in the options (the gain-loss formulation effect of Kahneman and Tversky, 1981).

In the present study, guilt influenced individual preferences, regardless of whether it was generated by the task to be solved or by other situations unrelated to the task. In general, our results are consistent with a growing body of evidence that different emotions of the same valence (e.g., fear, anger, or sadness) differentially influence judgments and choices (e.g., Giner-Sorolla, 2013; Lerner et al., 2015; Fitouchi et al., 2022). As expected, a novel aspect of our results is that individual choices are differentially influenced by different emotions of the same valence but both moral in nature, such as guilt and anger.

Furthermore, in contrast to previous studies (cf. Lerner and Keltner, 2000, 2001), we show that guilt does not lead to a specific preference for risky or riskless choices. In our studies, risky or riskless choices varied depending on which option led to the satisfaction of the guilt-activated goal, i.e., making amends or atonement (cf. Haidt, 2003; Mancini et al., 2008; Giner-Sorolla, 2013). Indeed, our participant’ preference shifted from a riskless choice (see results of Experiments 1 and 2 when the offender paid for the decision) to a risky one (see Experiment 2 when the victim paid for the decision), depending on which choice satisfied the moral goal involved. In contrast, according to the literature, anger always leads to a specific choice, the risky one, regardless of who pays for the decision or the framing effect. Thus, another original aspect of our results is that they cannot be explained by assuming only one form of bias that the different emotions exert on risky decisions.

Guilt creates a bias toward a riskless choice only when the offender has to pay for the choice. When, as in some of our experimental conditions, the person affected by the decision is the victim, the outcome is biased toward a risky choice. Instead, we suggest that a complex cognitive evaluation process (see Izard, 2009) is taking place in the background: the decision is mediated by the experimentally induced emotion and a cognitive evaluation of the possible outcomes. People experiencing guilt focus specifically on the negative consequences experienced by others (or themselves), thus promoting a motivation to “right the wrong” (see Gangemi and Mancini, 2021). According to this hypothesis, our participants’ behavior is driven by the goal of making amends and atonement (e.g., Haidt, 2003; Mancini and Gangemi, 2021; Tangney et al., 2007), for example, with reparative actions (e.g., confessions, apologies, or undoing the consequences of the behavior). In summary, moral emotions may have the adaptive role of balancing short-term utilitarian goals with long-term social goals (e.g., Castelfranchi, 2007). For example, we could hypothesize that in our first experiment, where offenders have to pay the fine, the utilitarian goal would lead them to make the choice that would minimize the payment (i.e., the monetary loss). However, avoiding the payment would likely lead to a form of social ostracism: the individual could be seen as someone who, having broken the rules, tries to avoid the punishment (Fehr and Gächter, 2002; Tabibnia and Lieberman, 2007; Rilling et al., 2008; Takahashi et al., 2009). Thus, there would be a trade-off between the short-term purely utilitarian goal and the long-term social goal of avoiding disapproval and punishment.

The induction of moral emotions, and in our case guilt, would have the effect of biasing a subject’s motivations toward the long-term social consequences by, as seen in our study, choosing the riskless choice, i.e., the choice that allows them to pay with certainty. The tendency of our perpetrators to make amends seems perfectly adaptive in the broader context of the social environment make amends seems perfectly adaptive in the broader context of the social environment.

## 4 Limits and future directions

In general, our study raises several questions. For example, why do our data contradict what we might expect from Lerner and Keltner’s (2001) study? Following Smith and Ellsworth’s (1985) theory of six cognitive dimensions underlying different emotions, Lerner and Keltner argued that fear and anger, although both negative, differ in terms of the dimensions of certainty and control. Whereas a sense of situational control and uncertainty defines fear, a sense of individual control and certainty defines anger. Accordingly, they found in their studies that fear and anger exert different influences on risk preference. More specifically, the sense of certainty and control associated with anger leads angry individuals to make risk-seeking choices across frames, whereas the sense of uncertainty and lack of control associated with fear leads fearful individuals to make risk-averse choices across frames. Since guilt is similar to anger in terms of the certainty and control dimensions (Smith and Ellsworth, 1985), in the current study we should expect that these two dimensions associated with guilt should lead guilty individuals to make risk-seeking choices across frames, analogous to anger. In contrast, as discussed above, we found that guilt did not lead to a specific preference for risky choices. The responsibility dimension, first introduced by Smith and Ellsworth (1985) and later replicated, extended and tested by other authors (e.g., Tesser, 1990; Ellsworth and Scherer, 2003), may help us to explain our data and answer our initial question. This dimension, defined as the extent to which one’s self or someone or something else is responsible for bringing about the event that arouses the emotion, seems to make a difference between anger and guilt. A strong sense of other responsibility is associated with anger, whereas a strong attribution of self-responsibility is associated with guilt, as also reported in recent studies on prosocial attitudes (e.g., Krettenauer and Jia, 2013). Thus, it seems that the sense of (self-)responsibility prevails over the dimensions of certainty and control, leading guilty individuals to make the choice (risky or riskless) that can satisfy the moral goal of restoring justice, by and repairing the damage caused to victim, expiating by the offense. This factor explains, for example, the data we observed in Experiment 2. When guilt was induced and the victim paid for the choice, then the choice was risky (i.e., the one that could give the victim a chance of obtaining justice, not paying at all). If the offender paid for the decision, then the choice was riskless (i.e., the one that made the offender pay with certainty). It would be useful to test in a future experiment whether the risky or riskless choices made by guilty participants to restore justice would lead to a pre-post decrease in the state of guilt.

A second question raised by this paper is why the moral emotion framing affects decision-making more than the descriptions of the outcomes provided in the options. A plausible answer may come from neuroscience. De Martino et al. (2006), using fMRI to investigate the neurobiological basis of the framing effect in a financial decision-making task, found that amygdala activation was significantly greater when subjects chose the safe option in the “win” frame and the risky option in the “lose” frame (the choices that constitute the framing effect). At the same time, in a between-subjects analysis, De Martino et al. (2006) found a negative correlation between individual susceptibility to the gain-loss framing effect and activity in the Orbitomedial Prefrontal Cortex (OMPFC): those subjects who were less susceptible to the effect showed greater activation in the OMPFC. Thus, on the one hand, the correlation between amygdala activation and the gain-loss framing effect supports the hypothesis that this bias is mainly driven by an affect heuristic modulated by the emotional system. On the other hand, the negative correlation between the gain-loss framing effect and OMPFC activation shows that this region, which is also involved in moral reasoning, has the power to counteract this bias. Other studies have obtained similar results (e.g., Roiser et al., 2009; Xu et al., 2013; Jepma and López-Sola, 2014). In particular, Xu et al. (2013) analyzed the neural basis of decision-making in people with trait anxiety using a decision-making task with frame manipulation (i.e., written description of options as potential gains or losses) and functional magnetic resonance imaging. The classic framing effect was observed: participants chose the safe option when it was described as a potential gain, but avoided the same option when it was described as a potential loss. Most importantly, trait anxiety was positively correlated with this behavioral bias. Trait anxiety was also positively correlated with activation of the amygdala-based “emotional” system and its coupling to the ventromedial Prefrontal Cortex (vmPFC) when decisions were consistent with the gain-loss framing effect, but negatively correlated with activation of the dorsal anterior cingulate cortex (dACC)-based “analytic” system and its coupling to the vmPFC when decisions were contrary to the gain-loss framing effect. Therefore, although abnormal activity in both amygdala (Tye et al., 2011) systems is usually associated with anxiety, indicating the potential influence of anxiety on frame-dependent decision making (Hartley and Phelps, 2012), Xu and colleagues’ findings instead suggest that trait anxiety is not associated with subjective risk preference. However, there is an evaluative bias of emotional information in decision-making, which is underpinned by a hyperactive emotional system and a hypoactive analytical system in the brain. According to these data, we can hypothesize that in the current study, the induction of specific emotions such as anger or guilt, which are moral in nature, would interfere with the gain-loss framing effect and prevail over a general emotional evaluation. However, further analysis is warranted to evaluate this hypothesis. Indeed, although we used a large sample of individuals (almost 550) to test our hypothesis, we should treat these results with caution and make further verifications with a larger sample.

## 5 Conclusion

Although there is sufficient evidence in the literature to move toward a general model of affective influences on decision making (Lerner et al., 2015; Fitouchi et al., 2022), emotion is not necessarily a form of heuristic thinking. Indeed, the distinction between the cognitive consequences of an emotion elicitation phase and an emotion persistence phase may be useful in linking emotions to modes of thinking. According to our study, guilt leads to both risky and riskless decisions, and thus to a more thoughtful evaluation of the situation. Therefore, our results confirm the hypothesis that guilt induction activates a dynamic emotional-cognitive evaluation process (as in Izard, 2009), capable of assessing the context and balancing short-term outcomes with possible long-term social consequences. Making amends or atonement for the offense may contradict a purely utilitarian perspective, but it is compatible with the social utility model and consistent with the motivation to avoid social ostracism and altruistic punishment by social peers. Making amends or atonement could reduce aversive guilt and activate the pleasure of acting fairly and cooperatively.

## Data Availability

The raw data supporting the conclusions of this article will be made available by the authors, without undue reservation.
